# Role of Hormonal Circuitry Upon T Cell Development in Chagas Disease: Possible Implications on T Cell Dysfunctions

**DOI:** 10.3389/fendo.2018.00334

**Published:** 2018-06-14

**Authors:** Ana Rosa Pérez, Alexandre Morrot, Vinicius Frias Carvalho, Juliana de Meis, Wilson Savino

**Affiliations:** ^1^Institute of Clinical and Experimental Immunology (IDICER-CONICET UNR), Rosario, Argentina; ^2^Faculty of Medicine, Tuberculosis Research Center, Federal University of Rio de Janeiro, Rio de Janeiro, Brazil; ^3^Oswaldo Cruz Institute, Oswaldo Cruz Foundation, Rio de Janeiro, Brazil; ^4^Laboratory of Inflammation, Oswaldo Cruz Institute, Oswaldo Cruz Foundation, Rio de Janeiro, Brazil; ^5^Laboratory on Thymus Research, Oswaldo Cruz Institute, Oswaldo Cruz Foundation, Rio de Janeiro, Brazil; ^6^National Institute of Science and Technology on Neuroimmunomodulation (INCT-NIM), Rio de Janeiro, Brazil

**Keywords:** Chagas disease, thymus atrophy, thymocytes, hypothalamus–pituitary–adrenal axis, growth hormone, prolactin

## Abstract

T cell response plays an essential role in the host resistance to infection by the protozoan parasite *Trypanosoma cruzi*, the causative agent of Chagas disease. This infection is often associated with multiple manifestations of T cell dysfunction, both during the acute and the chronic phases of disease. Additionally, the normal development of T cells is affected. As seen in animal models of Chagas disease, there is a strong thymic atrophy due to massive death of CD4^+^CD8^+^ double-positive cells by apoptosis and an abnormal escape of immature and potentially autoreactive thymocytes from the organ. Furthermore, an increase in the release of corticosterone triggered by *T. cruzi*-driven systemic inflammation is strongly associated with the alterations seen in the thymus of infected animals. Moreover, changes in the levels of other hormones, including growth hormone, prolactin, and testosterone are also able to contribute to the disruption of thymic homeostasis secondary to *T. cruzi* infection. In this review, we discuss the role of hormonal circuits involved in the normal T cell development and trafficking, as well as their role on the thymic alterations likely related to the peripheral T cell disturbances largely reported in both chagasic patients and animal models of Chagas disease.

## Introduction

Chagas disease, or American trypanosomiasis, is a tropical neglected illness caused by the hemoflagellate protozoan *Trypanosoma cruzi*. Chagas disease transmission to humans can be classified in primary (vectorial, blood transfusion, congenital, and orally) and secondary (less frequent, such as laboratory accident, handling of infected animals, organ transplantation from infected donors, and hypothetically through sexual) routes of *T. cruzi* infection (1, 2). Presently, oral transmission of human Chagas disease is the most important transmission route in the Brazilian Amazon region, mainly secondary to food/beverage contamination with *T. cruzi*. It is noteworthy that oral transmission has been associated with high mortality and morbidity, increased prevalence and severity of the cardiac pathology (myocarditis) (3–6). Argentina, Bolivia, Colombia, Ecuador, French Guiana, and Venezuela have also reported acute Chagas disease cases associated with contaminated food consumption (7–9).

*Trypanosoma cruzi* infection is presently considered as a world-wide health problem with deficiencies in treatment, absence of appropriated vaccines and world spreading (10, 11). The infection leads to an acute phase, with symptoms such as fever, muscle pain, swollen lymph nodes, hepatosplenomegaly, edema, tachycardia, dyspnea, pericardial effusion and inflammatory reaction at the vector’s biting site of the vector (chagoma) (2, 12). During the acute phase, circulating parasite numbers are systemically increased, and they are able to infect several tissues and cell types, such as skeletal and cardiac myocytes, macrophages, fibroblasts, neurons and epithelial cells. For this reason, amastigote nests were already described in glands, skeletal muscle, as well as, lymphoid and nervous tissues (11, 13). Following recovery from the acute phase, the patient enters into a long indeterminate, latent, phase with no symptoms and very low parasitism. The latent infection may remain silent for 10–30 years. One-third of infected patients in the latent phase develop clinical symptoms as chronic cardiac dysfunction (cardiomyopathy), megacolon or megaesophagus. It is believed that chronic chagasic cardiomyopathy has an autoimmune pathophysiological component, with involvement of T and B autoreactive cells, as well as promoted by the persistence of the parasite. At this phase, life expectancy decreases about 9 years in these clinical forms of chronic patients (14).

### T Cell Changes During *T. cruzi* Infection

In the immune system, *T. cruzi* infection promotes changes in the dynamics and in the size of T lymphocyte populations, contributing to regional response in primary, including thymus and secondary lymphoid organs (15). In infected mice, the thymus suffers a strong atrophy in the acute phase, due to massive death of CD4^+^CD8^+^ double-positive (DP) and CD4^+^Foxp3^+^ regulatory T cells (Treg) by apoptosis, accompanied by an abnormal escape of immature and potentially autoreactive T lymphocytes from the organ (11, 16). Interestingly, T cell abnormal escape was also documented in chronically *T. cruzi-*infected patients (17, 18). On the other hand, it is known that under physiological conditions, the re-entry of CD4^+^ and CD8^+^ T cells into the thymus is restricted to activated/memory cells (19), being driven by CCL2/CCR2 interactions (20). Some authors speculate that the re-entrance of T effector cells may influence the tolerance induction by promoting Treg development, since they represent the main source of IL-2 (21). Furthermore, Treg with a clear maturational phenotype were observed in the infected thymus, suggesting that they may correspond to peripheral Treg that have re-entered into the thymus (16). In any case, the physiological consequences of the Treg cell re-entry into the thymus remains undetermined.

Diverse groups have shown an expansion in secondary lymphoid organs such spleen and subcutaneous lymph nodes due to T and B cell polyclonal activation. In contrast, the mesenteric lymph nodes and Payer’s patches show atrophy and T lymphocyte death (15, 22–33).

An increase in IL-2 production is involved in subcutaneous lymph nodes hyperplasia in *T. cruzi* infection (15, 31). Spleen and subcutaneous lymph node hypertrophy is a consequence of tissue T/B lymphocyte activation and proliferation (15, 23, 31, 34, 35). Moreover, trans-sialidase, racemase, and *T. cruzi* DNA seem to contribute to T and/or B lymphocyte activation and cytokine production by interfering with interaction between dendritic cells and lymphocytes (36–40). In contrast to the hyperplasia seen in spleen and subcutaneous lymph nodes of infected mice, mesenteric lymph node atrophy is related to a local decrease in IL-2 and IL-4 production, with apoptotic death of T/B lymphocytes (15). In a second vein, it has been shown in the mouse model that splenectomy or mesenteric lymph node excision prior to *T. cruzi* inoculum increases susceptibility to infection, suggesting that these lymphocytes are involved in *T. cruzi* host immune response (15, 22–33).

## Systemic Hormonal Imbalance in Chagas Disease

Endocrine and immune systems control several physiological, biochemical, and functional activities in the organism both during homeostasis, including early development and aging (41), as in pathological situations, such as infectious and metabolic diseases (42, 43). Immunoendocrine interactions occur through bidirectional circuits, characterized by highly specialized signaling molecules known as cytokines and hormones, respectively (44). Given the extensive diversity of interactions between endocrine and immune cells, it is conceivable that disturbances of one or more of these components of the immunoendocrine axes lead to the development and/or exacerbation of several illnesses, including Chagas disease (42).

The hormonal imbalance in patients with Chagas disease has been discussed since the discovery and description by Carlos Chagas, who divided the symptomatology of chronic form of American Trypanosomiasis according to thyroid, heart, and central nervous system disease. In fact, the inclusion of the thyroid form of the disease was based on both clinical aspects, association of goiter with myxedema, and the detection of the parasite and inflammation in thyroid during autopsy (45). Currently, it is believed that Chagas disease by itself is not able to cause goiter, but may predispose patients to develop goiter (46).

One of the main endocrine circuits studied in Chagas disease is the hypothalamus–pituitary–adrenal (HPA) axis, since the release of glucocorticoid (GC) hormones is a protective mechanism of the host against the harmful effects of pro-inflammatory cytokines (47). Acute *T. cruzi* infection induces increased corticosterone levels in both C57BL/6 and BALB/c mouse strains (48), indicating a hyperactivation of HPA axis. Such an increase in circulating corticosterone levels is in close correlation with the hypertrophy of adrenal glands, including its *zona fasciculata*, and a rise in the expression of several steroidogenic enzymes, such as cytochrome P450, family 11, subfamily A, polypeptide 1 (CYP11A1), CYP11B1, 11β-hydroxysteroid dehydrogenase type 1 (HSD1), and steroidogenic acute regulatory protein (StAR) (49).

This HPA axis activation observed in experimental models of Chagas disease is associated with the presence of nests of *T. cruzi* amastigotes in the adrenals, as well as parasite-derived antigens in both adrenals and pituitary gland (50). Although by now, the underlyning mechanisms are not fully elucidated, the presence of *T. cruzi-*derived antigens (proteins, DNA, or glycolipids) in the endocrine glands of HPA axis may promote a local inflammatory response *via* the engagement of TLRs, as shown in bacterial models of infection (51). In particular, the stimulation of TLR-9, which recognizes *T. cruzi* DNA (52), may cause the local production of cytokines and consequent increase in the release of corticosterone, as seen in a model of sepsis (53). Similarly, TLR-2 or TLR-4 pathways might be stimulated by TLR agonists expressed by *T. cruzi* like GPI or GIPL anchors, respectively (54). In fact, C57BL/6 mice infected with *T. cruzi* showed, not only in plasma but also intra-adrenal, increased levels of TNF-α, IL-1β, and IL-6 (55), suggesting that these proinflammatory cytokines are involved in the hyperactivation of HPA axis at different levels.

Although infected mice presented the parasite and a pronounced inflammatory response in the pituitary gland, the systemic levels of adrenocorticotropic hormone (ACTH) are not changed (49, 50), suggesting that the increase in circulating corticosterone levels noted in infected mice occurs independently of ACTH. In fact, both systemic and intra-adrenal cytokine production may favor adrenal inflammation during infection, which can directly trigger and sustain an alternative way of adrenal secretion of GC, resulting uncoupled from the hypothalamic–pituitary unit (56). Structural alterations like vascular changes within the endocrine microenvironment may also lead to a transient HPA dysfunction (56). Also, local inflammation driven by the presence of *T. cruzi* or their antigens may promote the income of inflammatory cells. Strikingly, adrenals of infected mice showed leukocyte infiltration, characterized by the presence of CD8^+^ and CD4^+^ T lymphocytes, as well as macrophages and enhanced expression of extracellular matrix (ECM) deposition, including fibronectin and laminin (44). These ECM molecules might fix pathogen-derived antigens as well as pro-inflammatory cytokines released during immune response, thus contributing to the establishment of inflammation and sustaining GC production (56).

Pituitary hormones, including growth hormone (GH) and prolactin (PRL), act as modulators of the immune system (57, 58). Similarly to GC, GH and PRL are considered stress-related hormones (59, 60), having opposing actions of GC on the viability and proliferation of thymic cells (61). In GH-/PRL-secreting GH3 cells, the infection with *T. cruzi in vitro* induces a reduction in the secretion of both GH and PRL (62). These results suggest that *T. cruzi* infection decreases GH and PRL production by the pituitary. In fact, chagasic patients showed decreased GH levels in response to glucose or insulin compared to healthy subjects (63), and mice infected with *T. cruzi* presented a reduction in plasma PRL levels (64). In effect, the low production of PRL by pituitary induced by *T. cruzi* infection seems to directly affect the high corticosterone synthesis by the adrenals (65). Interestingly, while asymptomatic patients showed a tendency to diminish the secretion of GH, individuals with severe cardiomyopathy show increased levels of this hormone and also an altered GH/IGF-1, suggesting an imbalance in this axis (65).

Besides GC and pituitary hormones, some gonadal steroid hormones, including dehydroepiandrosterone-sulfate (DHEA-s) and testosterone, can be altered in human or experimental Chagas disease (66, 67). Animals infected with *T. cruzi* presented a reduction in serum testosterone levels in the acute phase of infection. However, histological analyses in testes, seminal vesicles, and epididymis did not reveal any differences between control and infected animals (68). In addition, *T. cruzi-*infected animals showed an increase in circulating levels of estradiol (67). Regarding DHEA-s levels, rats infected with *T. cruzi* did not alter the DHEA-s systemic levels. However, chronic chagasic patients with different degrees of myocarditis presented a marked reduction in DHEA-s levels. Interestingly, although the alterations in the levels of DHEA in animals are not seen in patients with Chagas disease, both animals and patients presented an increase in GC/DHEA-s ratio, which is important for the development of an anti-inflammatory milieu (66, 67).

## Hormones and Their Relationship with Thymus Atrophy in *T. cruzi* Infection

T cell response plays an essential role in the host resistance to the *T. cruzi* infection, but sub-clinic and clinic manifestations of Chagas disease can be associated with multiple manifestations of T cell dysfunction (69–73). Additionally, as seen in animal models of Chagas disease, there is a strong thymic atrophy characterized by loss of thymus weight, massive death of CD4^+^CD8^+^ DP cells by caspase-dependent apoptosis (32), alterations in the double-negative (DN) T-cell population (74, 75), depletion of thymic Treg (16) and also an abnormal and premature escape of immature and potentially autoreactive DP and DN thymocytes from the organ (17, 26, 74, 76). Furthermore, it has been recently described that during experimental *T. cruzi* infection, bone marrow aplasia and a diminution in common lymphoid progenitors appear before thymic alterations (75).

Due to the possible autoimmune component of chagasic myocarditis, it is plausible to hypothesize that thymic selection mechanisms could be altered as a consequence of the infection. In this regard, in BALB/c mice, some T-cell receptor (TCR) Vβ families, which under normal conditions should have undergone negative selection through apoptosis, appear in the periphery of the immune system during *T. cruzi* infection and might potentially conduce to autoimmune reactions (77). Nevertheless, in the same study, potentially autoimmune mature T cells were not seen within the thymus. Using an (OVA)-specific TCR transgenic system, we confirmed that the negative selection process is normal during experimental *T. cruzi* infection. In addition, the expression of autoimmune regulator factor (AIRE) expression and tissue-restricted antigen genes were normal in the thymus of infected animals (17). However, similarly to what is described in the murine model, activated DP T cells with an activated phenotype are found in the blood of patients with chronic Chagas disease in association with severe myocarditis (17), suggesting that some intrathymic checkpoints might be failed. This may have related to T cell trafficking alterations due to changes in the patterns the ECM protein deposition within the organ, expression of ECM receptors on thymocytes and thymic Tregs, as well as changes in cell migration-related cytokines (16, 32, 78, 79).

Normal T cell development is tightly controlled not only by cell–cell interactions and cytokines, but also by hormones, interacting *via* a diversity of endocrine and paracrine pathways, acting on thymocytes and thymic microenvironmental cells *via* specific receptors (42, 80). Moreover, thymic cells not only respond to systemic levels of hormones but also constitutively synthetize and secrete hormones locally, such as GC, GH and PRL. In this context, disturbances in hormone levels caused by inflammation can interfere with the normal T cell development. Accordingly, increased evidence indicates that the thymic alterations seen during *T. cruzi* infection are strongly associated to hormonal imbalance, involving systemic or intrathymic axes.

### The HPA Axis

It is well known that, if not controlled, systemic effects of GC on the adaptive immunity can promote immunological disturbances. The HPA axis activation, through the production and action of GC, plays a major role in protecting the host against the inflammatory acute stress caused by *T. cruzi* infection (48, 55). Nevertheless, immature DP thymocytes are major targets of HPA axis activation, since enhanced levels of GC seen in experimental acute *T. cruzi* infection induce DP thymocyte depletion through caspase-dependent apoptosis (32, 81). In this regard, blockade of GC receptor activity with RU486 prevented DP thymocyte apoptosis (48, 55) together with caspase-8 and caspase-9 activation (32). Interestingly, both thymic epithelial cells and DP thymocytes can also synthetize GC, suggesting that both paracrine and autocrine loops influence thymocyte survival during *T. cruzi* infection (82, 83). In addition, *T. cruzi* is able to infect thymic epithelial cells (84), indicating that the parasite *per se* may alter the local production of hormones and determining thymocyte fate. Yet, this hypothesis needs experimental confirmation.

### GH and PRL

Prolactin is not only produced in the anterior pituitary gland but also in a range of tissues including adipose tissue, skin, and thymus. Actually, both GH and PRL exert relevant roles upon thymus physiology and are constitutively produced and secreted by thymocytes and thymic epithelial cells (TEC) (85–87). Increased intrathymic expression of GH leads to an enlarged thymus, as can be observed in transgenic mice that overexpress the hormone or in individuals treated with recombinant forms of GH (88–90). In addition, GH and IGF-1 (the hormone that mediates several GH effects) favor thymocyte migration, augmenting ECM deposition (85). Moreover, specific receptors for GH, IGF-1 and PRL are constitutively expressed by TEC and thymocytes, indicating autocrine and paracrine regulatory loops, in addition to the systemic effects of these hormones (57, 90).

The action of these anti-stress hormones is actually one of the ways that counterregulate systemically or in an organ-specific fashion, the action of the GC produced during *T. cruzi* infection. We have shown that PRL plays a critical role in balancing the effects of corticosterone in the thymus during *T. cruzi* infection (65, 74). In the mouse model of *T. cruzi* acute infection, we found an intrathymic cross-talk between GC receptors (GR) and PRL receptors that seems to work to counteract the effects of the infection, toward the neutralization of GC-related systemic deleterious effects on DP thymocyte survival during parasite-induced thymic atrophy. Furthermore, we showed that injection of metoclopramide (known to enhance PRL secretion by the pituitary gland), during experimental infection, preserved the thymus from atrophy during infection with *T. cruzi* (65). This event was associated with partial prevention of DP thymocyte apoptosis as well as thymic release of undifferentiated and potentially autoreactive DP cells to the peripheral lymphoid tissues. These findings point to a modulation of the stress-related hormonal circuits in the intrathymic T cell development during *T. cruzi* infection.

### Testosterone and DHEA

Androgens in general, and especially testosterone, have immunosuppressive actions on the immune system, whereas the androgen DHEA seems to have immunostimulating properties, and counteracts the immunosuppressive effects of GC (91). In a second vein, it is widely accepted that sexual dimorphism is strongly related with differences in immune function and disease outcome. Concerning experimental Chagas disease, females are more resistant to infection than males, and androgen depletion improved the resistance against *T. cruzi* (92–94). Interestingly, in male mice, DP thymocyte death within thymic nurse cells seems to be caused by testosterone (95) and testosterone supplementation causes a diminution in thymocyte proliferation (96). Unlike GC, known to activate caspase-8 and caspase-9-mediated apoptosis in thymocytes, testosterone is able to activate thymocyte apoptosis through a caspase-3-dependent pathway (95). Studies in the rat model of *T. cruzi* acute infection revealed that DHEA supplementation promotes thymocyte proliferation, suggesting that DHEA treatment may prevent DP loss and other thymic alterations (96). Nevertheless, more studies are needed to evaluate the role of sex hormones in the thymic atrophy that occurs during *T. cruzi* infection.

## Metabolic Alterations and Adipokines

In parallel to the endocrine imbalance, animals infected with *T. cruzi* also show a clear metabolic disturbance, including hypoglycemia, weight loss and leptin alterations (97). It is known that, besides controlling saciety, leptin plays protective affects upon intrathymic T cell development under physiologic conditions (98, 99). Nevertheless, in acute *T. cruzi-*infected C57BL/6 mice, its systemic and adipose tissue derived-expression is sharply diminished, suggesting that its loss may fuel thymic atrophy (97) However, and unlike what happens in models of experimental endotoxemia (100), leptin replacement during the acute infection, while attenuating GC release, fails in reversing thymic atrophy (97). The reason of this difference should be investigated, but it is possible to speculate that thymic ObR expression during *T. cruzi* infection could be also diminished, as previously observed at the hypothalamic level (97). In this regard, when infected db/db mice (that lack ObR) were reconstituted with the brain ObR, the infection was less obvious (101). These data suggest that leptin axis is dysregulated during infection. Strikingly, in chronic obese model of infection and also in human chronic disease, it was reported that adipokine disturbances are related to myocardial damage and heart autonomic dysfunction (102, 103), while their effects upon T cell dynamics has not been estimated.

## Conclusion

**Figure 1:**
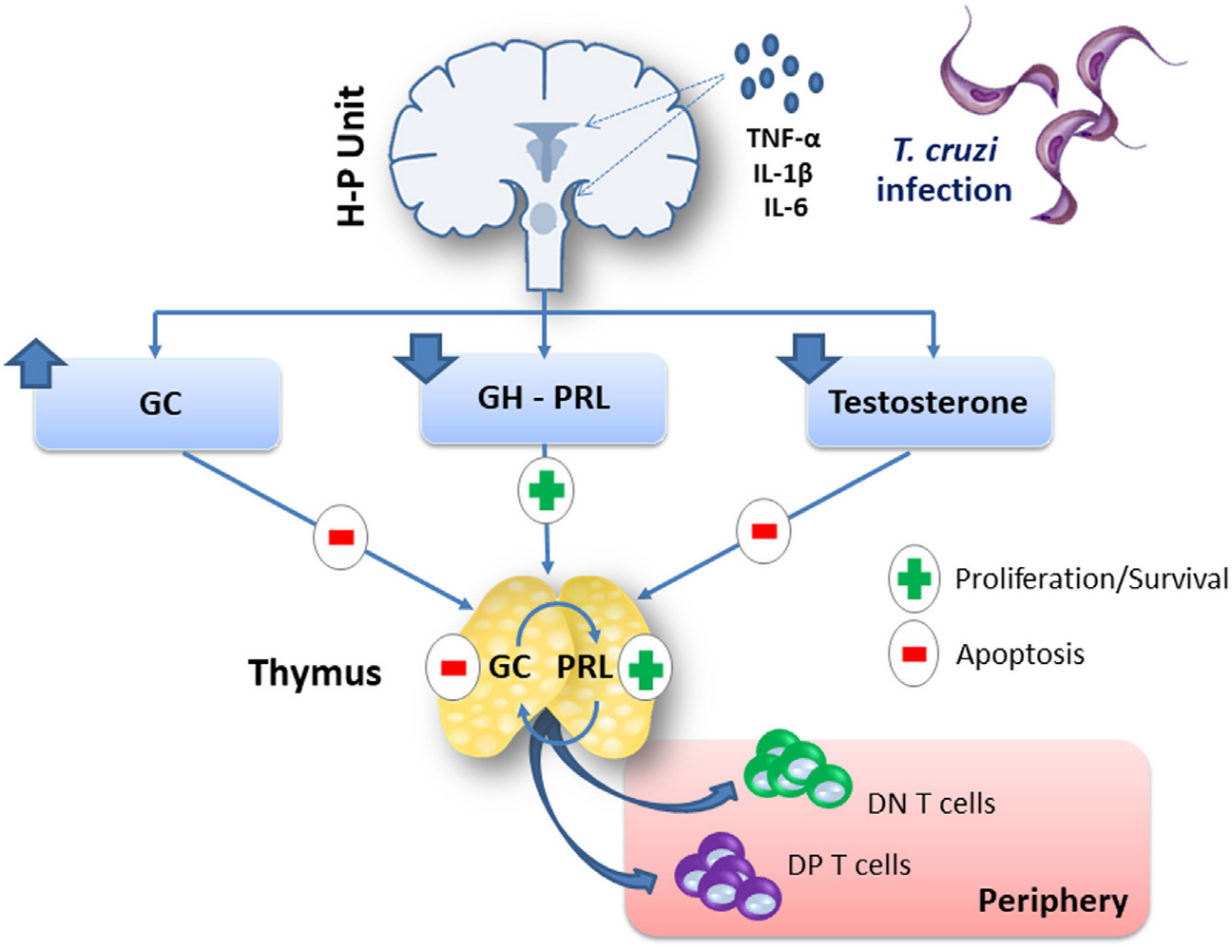
Systemic and intrathymic hormonal imbalance affects the thymus during experimental *Trypanosoma cruzi* infection. Acute *T. cruzi* infection in mice induces a rise in plasma levels of proinflammatory cytokines, which are involved in the hyperactivation of the hypothalamus–pituitary–adrenal (HPA) axis. Pro-inflammatory cytokines can enhance HPA axis activation, by acting at the hypothalamus–pituitary unit and/or on peripheral glands, i.e., the adrenals. *In situ* inflammatory reactions caused by *T. cruzi*-derived antigens or structural changes like vascular alterations or an enhanced extracellular matrix deposition in the endocrine microenvironment may also lead to sustain glucocorticoid hormone (GC) levels. The increment of systemic and intrathymic GC levels causes thymic atrophy by depletion of CD4^+^CD8^+^ double-positive (DP) thymocytes through apoptosis. In parallel, there is an abnormal export of immature DP and double-negative (DN) T cells to the periphery of the immune system. Growth hormone (GH) and prolactin (PRL) have positive effects upon the thymus, but *T. cruzi* infection decreases GH and PRL production by pituitary cells. Male animals acutely infected with *T. cruzi* also present a reduction in serum testosterone levels, although DP thymocyte death seems to be induced by this androgen, whereas testosterone supplementation induced a diminution in thymocyte proliferation. Abbreviation: H–P unit, hypothalamus–pituitary unit.

There is no doubt that acute *T. cruzi* infection induces an immunoendocrine imbalance, which somehow favors the ability of the parasite to settle in the host, and the development of distinct pathological events, among which the massive thymocyte death and consequent thymic atrophy. Yet, this is a complex network of events (summarized in Figure 1 and Table 1) that needs further investigation, including the possibility of endocrine axes being target for complementary therapeutic intervention in Chagas disease.

**Table 1:** Effects of hormonal imbalance upon thymocytes during *Trypanosoma cruzi* infection.

	GC	DHEA	PRL	GH	Testo	Leptin (*)	Reference
Weight/size	↓	↑	↑	↑	↓	↓	(18, 42, 44, 45, 53, 104)
Cellularity	↓	↑	↑	↑	↓	↓	(25, 42, 44, 46, 54, 55, 66, 73, 88, 104)
Apoptosis of DP cells	↑	↓	↓	↓	↑	↓	(25, 42, 44, 46, 54, 55, 73, 87, 88, 104)
Loss of Tregs	↑	ND	ND	ND	ND	ND	(66)
Vβ T-cell repertoire/negative selection	ND	ND	ND	ND	ND	ND	(19, 67)
Altering intrathymic cell migration	ND	ND	ND	ND	ND	ND	(21, 66, 69, 80)
Escape of DP/DN cells to periphery	ND	ND	↓	ND	ND	ND	(11, 55, 64, 67, 89, 105)

DP, CD4^+^CD8^+^ double-positive; GC, glucocorticoids; DHEA, dehidroepiandrosterone; PRL, prolactin; GH, growth hormone; Testo, testosterone; ND, not determined; ↑, increase; ↓, decrease; (*) effects caused by administration.

## Author Contributions

All authors contribute equally to the manuscript: AP, AM, VC, JM, and WS.

## Conflict of Interest Statement

The authors declare that the research was conducted in the absence of any commercial or financial relationships that could be construed as a potential conflict of interest. The handling editor is currently co-organizing a Research Topic with the authors AP, WS and confirms the absence of any other collaboration.
